# Influence of Socio-Economic Factors and Region of Birth on the Risk of Preeclampsia in Sweden

**DOI:** 10.3390/ijerph19074080

**Published:** 2022-03-30

**Authors:** Kristina Mattsson, Sol Juárez, Ebba Malmqvist

**Affiliations:** 1 Division of Occupational and Environmental Medicine, Institute of Laboratory Medicine, Lund University, SE-223 83 Lund, Sweden; ebba.malmqvist@med.lu.se; 2 Centre for Health Equity Studies (CHESS), Department of Public Health Sciences, Stockholm University, SE-114 19 Stockholm, Sweden; sol.juarez@su.se

**Keywords:** socio-economic factors, preeclampsia, region of birth, immigrant health, pregnancy health

## Abstract

Objectives: To investigate the association between socio-economic factors and the risk of preeclampsia in Sweden, specifically investigating if this relationship is confounded by maternal region of birth. Study design: All singleton births between 1999 and 2009 in an ethnically diverse area in southern Sweden, totaling 46,618 pregnancies, were included in this study. The data on maternal pregnancy outcomes were retrieved from a regional birth register and socio-economic variables from Statistics Sweden. The risk ratios for preeclampsia were calculated for educational level and household disposable income, adjusting for maternal region of birth, maternal age, body mass index, parity, and smoking. Results: Low income levels were associated with a higher risk for preeclampsia, adjusted risk ratio (aRR) = 1.25 (95% confidence interval [CI]: 0.99, 1.59) and aRR = 1.36 (95% CI: 1.10, 1.68) for the two lowest quintiles, respectively, compared to the highest. There was an educational gradient in preeclampsia risk, although not all categories reached statistical significance: aRR = 1.16, (95% CI: 0.89–1.50) for low educational attainment and aRR = 1.23 (95% CI: 1.08, 1.41) for intermediate educational attainment compared to women with highest education. The socio-economic gradient remained after adjusting for region of birth. There was a lower risk for preeclampsia for women born in Asia, aRR = 0.60 (95% CI: 0.47, 0.75), regardless of socio-economic position. Conclusion: An increased risk for preeclampsia was seen for women with measures of lower socio-economic position, even in a universal, government-funded healthcare setting. The relationship was not explained by region of birth, indicating that the excess risk is not due to ethnically differential genetic pre-disposition but rather due to modifiable factors.

## 1. Background

Preeclampsia is one of the most common maternal morbidities during pregnancy, estimated to affect 2–8% of all pregnancies worldwide [1]. In spite of the significant clinical and public health problem posed by preeclampsia, much remains unknown about the condition.

In Sweden, where implementations have been made to make maternal health care universal and accessible to all pregnant women, studies report socio-economic disparities in the risk for adverse pregnancy outcomes, where immigrant women or women with lower socio-economic position have been shown to be at increased risk [2,3,4]. Similar findings have been reported from other countries with universal health care [5]. It is still unclear, however, if there is an association between socio-economic factors and preeclampsia, as previous studies report conflicting results. Socio-economic measures that have been reported to increase the risk for preeclampsia include social deprivation [6], low income levels [7], low educational attainment [8], and receiving governmental medical aid [5]. At the same time, other studies have failed to link socio-economic factors with preeclampsia or other hypertensive disorders during pregnancy [9,10,11,12,13,14] have found that the associations were mediated by maternal BMI [15,16], or even report preeclampsia to be more common among women from socio-economically more affluent areas [17].

Furthermore, considering that previous studies have shown an ethnic variation in the incidence of preeclampsia [11,18], and being foreign-born potentially influences the woman’s socio-economic position, it is of importance to first investigate if there are socio-economic determinants influencing preeclampsia risk and, secondly, to elucidate if such a gradient is confounded by region of birth, potentially increasing preeclampsia risk via, e.g., genetic predisposition. Indeed, other studies have highlighted the complex, geographically dependent, relationship between region of birth/immigrant status, socio-economic factors, and perinatal outcomes [19,20,21].

Hence, the study had two aims. First, to examine whether socio-economic factors, measured as maternal educational level and household disposable income, are associated with the risk of preeclampsia. The second aim was to investigate if the maternal region of birth influences preeclampsia risk, and whether it confounds the relationship with socio-economic factors.

## 2. Materials and Methods

### 2.1. Data Sources and Study Population

The study population comes from previously established pregnancy cohort, The Maternal Air Pollution in Southern Sweden (MAPSS). The cohort includes virtually all singleton births between 1999 and 2009 in the Malmö–Lund–Trelleborg area in southern Sweden. It comprises more than 48,000 births and was constructed by linking a regional birth register, Perinatal Revision South, with socio-demographic variables from Statistics Sweden. All individuals in Sweden are assigned a 10-digit personal identification number at birth, enabling the linkage between different data sources.

The Perinatal Revision South is a population-based regional birth register established in 1994, covering the whole of southern Sweden, with the aim of healthcare quality surveillance within obstetrics and perinatal care. All hospitals in the region with delivery units report to the register, and reporting is based on copies of standardized medical journals used during maternal healthcare at birth and the immediate neonatal period. In Sweden, home births are rare, maternal healthcare is free, and almost all pregnant women attend, allowing for very high register coverage.

**Figure 1:**
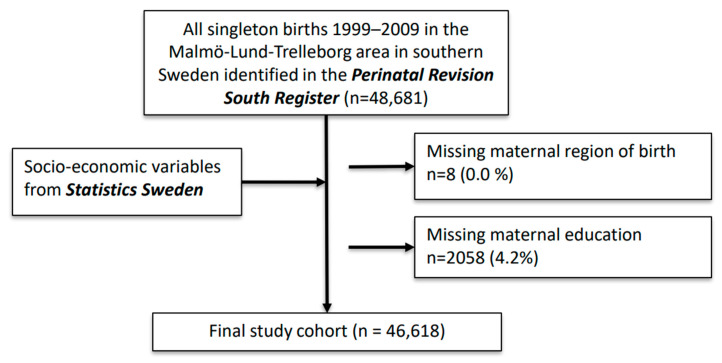
Flowchart depicting register linkage and how the final sample was reached.

Figure 1 depicts the register linkage and how the final sample subsequently was reached.

### 2.2. Outcomes

Women with preeclampsia were noted in the register by ICD-10 codes O14.0, O14.9, and O11.9, corresponding to the definitions of preeclampsia for that time: systolic blood pressure ≥140 mmHg and/or diastolic blood pressure ≥90 mmHg (measured twice with at least 6 h in between) and proteinuria >0.3 g/day, after 20 weeks of gestation. The diagnostic criterion for severe preeclampsia was blood pressure ≥160/110 mmHg or proteinuria ≥5 g/day. There were 1383 (2.8%) women with preeclampsia, and 236 (0.5%) of them were classified as severe (ICD-10 codes: O14.1 and O15). No distinction between early- and late-onset preeclampsia is made within the register.

### 2.3. Exposure Assessment—Socio-Economic Measures

Information on socio-economic determinants and region of birth was retrieved from the LISA database (Longitudinal integrated database for health insurance and labour market studies) and the Total Population Register, administered by Statistics Sweden. We retrieved household disposable income (including all social benefits such as sick leave and parental leave) for the year of the birth of the baby. Individuals ≥18 years with common children sharing place of residence are considered constituting a household in the registers, regardless of marital status. However, unmarried couples expecting their first child are not registered as a household, and thus the income levels for unmarried primiparous women are based solely on her income. We divided household disposable income into quintiles based on income levels for the total study population, provided the registered income was ≥SEK 80,000 (roughly corresponding to levels of social welfare, excluding rent) [22]. The women with taxable income below this threshold (*n* = 2401, 4.9%) were included in the analyses as a separate category.

The highest maternal educational level was retrieved for the year of the birth of the child and divided into (i) primary education (≤9 years of schooling; 13% of study population), (ii) secondary education (≤12 years of schooling; 41%), and (iii) post-secondary education/university level (42%). Statistics Sweden collects data on maternal education mainly from the Register of Education. For the women who did not complete their education in Sweden (mainly foreign-born), the information is collected from additional sources (e.g., from postal questionnaires, the public employment service, and the Migration Register). For 2058 (4.2%) women, there were no data on registered education.

Statistics Sweden does not provide country-specific data on maternal region of birth, but it is proxied by the following groups: 1. Sweden, 2. other Nordic country, 3. other country in the European Union, 4. other European country (non-EU), 5. North America, 6. South America, 7. Africa, 8. Asia, 9. Oceania, and 10. the former Soviet Union. Due to small numbers in several of the categories, we aggregated the data into Sweden, Nordic countries, Europe including former Soviet Union, the Americas, Asia and Oceania, and, lastly, Africa. We excluded *n* = 8 women with unknown or no region of birth registered.

### 2.4. Covariates

**Figure 2:**
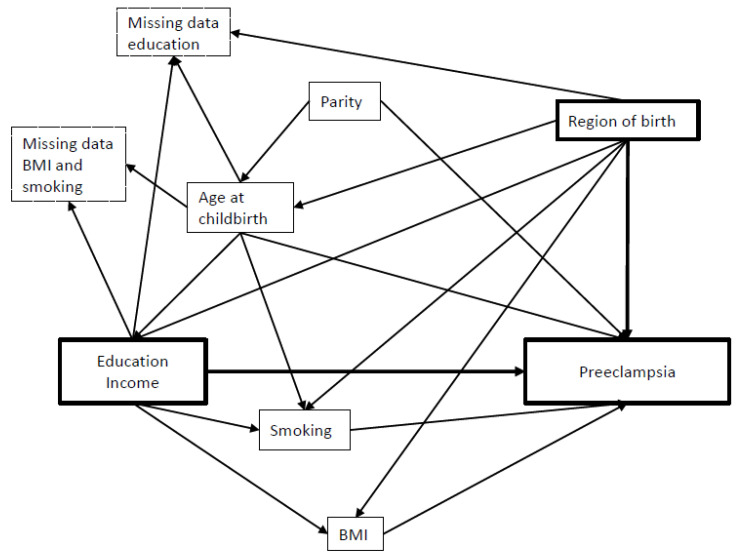
Directed Acyclic Graph conceptualizing the relationship between variables.

Figure 2 depicts a Directed Acyclic Graph conceptualizing the relationship between our exposures and outcomes of interest, as well as with potential confounders. The following covariates were considered confounders: maternal age at childbirth (four categories: <25, 25–29, 30–34, and ≥35 years), parity (3 categories: 1, 2, and ≥3), normal weight vs. overweight/obesity in early pregnancy (i.e., body mass index (BMI) <25 and ≥25 kg/m^2^), and maternal smoking during pregnancy (dichotomized: non-smoker, smoker).

#### Missing Data

A total of 8 women with missing data on region of birth and *n* = 2058 (4.2%) women with missing data on educational level were excluded from the study, as missingness for education was not at random (missingness associated with region of origin, age, as well as a diagnosis of preeclampsia). Thus, imputing values for education might introduce bias [23]. Characteristics of women excluded due to missing data are shown in Supplementary Table S1. Among the remaining women, there were *n* = 5645 (12.1%) women with missing data on BMI and *n* = 3327 (7.1%) on smoking during pregnancy. Having missing data on BMI and smoking was associated with being younger, being born outside of Sweden, and having lower educational attainment but not with the outcome. Missing data for BMI and smoking were imputed using multivariate imputation by chained equations with a generalized logit distribution (50 imputations).

### 2.5. Statistical Analyses

To describe differences between the populations according to preeclampsia status, chi-square tests and Mann–Whitney U (Wilcoxon) test were used. Risk ratios for preeclampsia by socio-economic variables and region of birth were calculated using a log-binomial regression model, generating risk ratios (RR) and 95% confidence intervals (CI). Dependence between data due to multiple pregnancies within the same woman was accounted for by adding a cluster term to estimate robust standard errors.

Initial models investigated maternal education and income separately, after which we included the following set of potential mediators: maternal age, BMI, parity, and smoking during pregnancy. To evaluate the possibility that region of birth confounds the association, we further included region of birth in the model. We also performed analyses with region of birth as the determinant of interest, subsequently adjusting for socio-economic measures and the confounders above.

For income, we performed a subset of analyses including only multiparous women to account for potential misclassification regarding unmarried cohabiting women not registered as a household.

The statistical analysis was performed using SAS version 9.4 (Copyright c 2013 by SAS Institute Inc., Cary, NC, USA).

## 3. Results

The basic characteristics of the cohort are shown in Table 1. In total, 46,618 women were included in the analyses, of which *n* = 1345 were diagnosed with preeclampsia. Women who developed preeclampsia were more likely to be primiparous, obese, and non-smokers (*p*-value for all comparisons <0.001).

The crude and adjusted risk ratios for preeclampsia by different levels of maternal education and income are shown in Table 2. There was a higher risk to develop preeclampsia for women with an intermediate level of education as well as for lower income levels. These associations remained after region of birth was included in the model (Table 2). The lowest level of education and income also showed a trend towards an increased risk, but the estimates did not reach statistical significance due to fewer women in these groups (Table 2).

There was a statistically significant decreased risk for women from Asian countries, adjusted RR = 0.60 (95% CI: 0.47, 0.75), but all other regions of birth showed similar risks as for Swedish-born women (Table 3).

## 4. Discussion

In these population-based data covering all singleton births in a diverse area of southern Sweden, a socio-economic gradient in the risk of preeclampsia was revealed, where women with lower income levels and lower educational attainment were at higher risk to develop the disease. The gradient was stable after adjustment for the maternal region of origin, indicating that the excess risk does not seem, at large, to be driven by a differential genetic pre-disposition, although we did find that women from Asian countries were at lower risk to develop preeclampsia regardless of socio-economic position.

Our study has several strengths. The use of population-based register data, collected routinely within maternal healthcare and including virtually all singleton births within a diverse uptake area, makes the risk of selective participation unlikely. Efforts have been implemented to make maternal healthcare in Sweden accessible and equal, e.g., it is completely free of charge. Attendance rates are very high (with only 1‰ abstaining completely from participating) [24], which is why we believe that most women with preeclampsia are included in our study cohort. To best capture the socio-economic context, multiple measures were used in this study, which is another strength. Education is feasibly measured and has been argued to be a good indicator of socio-economic factors over the life-course [25]. It is presumed to capture a variety of health-relevant traits such as life-style choices, intellectual and material resources, and health literacy and is a strong determinant of future income and employment [25]. Even though the registers only document completed education, the mean age of childbirth in this cohort was 30 years (standard deviation 4.9 year), meaning that the majority of the women likely had finished their education. There is a potential risk for some misclassification among immigrant women as they are asked to report their education by mail to Statistics Sweden, but the results were also robust when income levels were used as the socio-economic indicator.

Social deprivation [6], low income levels [7], low maternal educational attainment [8], and receiving governmental medical aid [5] have all been reported to confer an increased risk for preeclampsia. On the other hand, several publications report no link between different socio-economic factors and the development of preeclampsia or other hypertensive disorders during pregnancy [9,10,11,12,13] or have found that the associations were largely mediated by pre-pregnancy BMI [15,16]. It should be noted, however, that the studies reporting no association were either (i) based on samples of only a couple of hundred cases of preeclampsia, and hence might not have had sufficient power to detect any associations [9,10,12], or (ii) were based on a dichotomized measure of education (less vs. more than high school), and hence potentially lacking granularity to detect any difference [11,13]. One Swedish study found preeclampsia to be more common among women from higher socio-economic groups, although the authors speculate that this might be due to the a higher proportion of primiparas in the more affluent areas [17].

The current study is an addition to previous studies, many of which lacked information on maternal region of birth [7,9,10,15] or had other methodological limitations, including an exposure assessment based on the spouse’s socio-economic factors [10] or residential context [17], as well as small study samples [6,7,8,9,10,12,15,17]. Additionally, selection bias cannot be ruled out from studies requiring participation in cohorts [8,12,15].

With one exception, we did not find a differential risk in preeclampsia for the other regions of births compared to Swedish-born women. However, it should be noted that the power to detect such differences might have been low for some regions due to few women in these groups. The lower risk for preeclampsia for women born in Asian countries is in line with a previous report [18]. A recent review reported a pooled estimate of a 26% reduction in the risk for pregnancy-related hypertension for immigrants generally, suggesting a healthy migrant effect [26], whereas another review found indications of a healthy migrant effect in birth outcomes in an U.S. context, but not when European countries were the receiving countries [20]. We did not find an increased risk for women born in African countries, which has been reported earlier in an American context [18]. The reasons for this could include difference in life-style factors and obesity rates between African immigrant women in Sweden compared to African-American women in the United States. Another possible explanation could be access to healthcare services, both during and before pregnancy, potentially influencing other risk factor for preeclampsia. Even though the small group that do not attend maternal healthcare in Sweden to a higher degree are immigrant women [24], the general participation rate is very high, and healthcare services are free of charge.

There are limitations in this study that warrant further discussion. We did not have access to country-specific data on maternal region of origin, and due to small numbers in some of the groups, we had to group women in relatively large geographical regions of origin, rather than stratification by, e.g., low- vs. high-income countries. Consequently, the power to detect associations between preeclampsia and region of birth was low. In addition, different immigrant groups that have been shown to fare differently well in Sweden might have been analyzed together [2]. Furthermore, the women’s immigrant status (refugees vs. labor migrants) might also be a source of heterogeneity, as suggested in one previous study that used certain countries of birth and year of arrival as a proxy for having refugee status [27]. Although we do not have information on the reason for migration, a study revealed that the administrative reason (as opposed to probably the actual reason) is not relevant to identify immigrant women at higher risk of experiencing adverse reproductive outcomes [28]. We believe that our classification might to some extent account for this variation, however, as most non-European migrants in Swedes come from countries in conflict. Unfortunately, as most studies in this field, we were not able to identify undocumented migrants from the registers.

Unmarried couples expecting their first child are not registered as a household by Statistics Sweden; the income levels for these women are based on maternal income only, which might have underestimated the income level for some women. After the birth of the first child, the parents are considered a household regardless of marital status if they share place of residency. We believe this would be less of a problem for women born outside Sweden, as studies have shown that they tend to marry to a higher extent and at an earlier age [29]. In addition, analyses restricted to multiparous women did not change the results.

Some clinical information known to be relevant for preeclampsia risk was lacking, such as certain maternal pre-existing conditions, twin pregnancies, and the use of assisted reproduction. However, these factors are, at large, not associated with socio-economic position in Sweden (e.g., in vitro fertilization is subsidized and free of charge the first three attempts) and would likely not have altered the results. Lastly, the gestational week of preeclampsia onset is not recorded in the register, which precluded any analyses stratified by early and late onset preeclampsia, respectively.

## 5. Conclusions

In summary, the findings in this study indicate that socio-economic factors contribute to the development of preeclampsia and that the differential risk likely is not solely a reflection of maternal region of birth, although data granularity was too low to permit detailed analyses on maternal origin. This implies an excess risk that is potentially modifiable and further studies should investigate if maternal health in vulnerable sub-groups can be improved by directed efforts within maternal healthcare.

## Figures and Tables

**Table 1:** Characteristics of women included in study population, numbers in *n* and (%).

	Reference Women	Preeclampsia
Women total *n* = 46,618	*n* = 44,298	*n* = 1345
Maternal age at childbirth (years)		
<25	6256 (14.1%)	198 (14.7%)
25–29	13,850 (31.3%)	446 (33.2%)
30–34	15,771 (35.6%)	424 (31.5%)
35–39	7145 (16.1%)	221 (16.4%)
≥40	1276 (2.9%)	56 (4.2%)
Parity		
0 (primipara)	20,543 (46.4%)	937 (69.7%)
1	15,476 (34.9%)	261 (19.4%)
≥2	8279 (18.7%)	147 (10.9%)
Pre-pregnancy BMI (kg/m^2^)		
Missing	5342 (12.1%)	193 (14.3%)
<18.5 (underweight)	1056 (2.4%)	17 (1.3%)
18.5–<25 (normal weight)	24,755 (55.9%)	537 (39.9%)
25–<30 (overweight)	9416 (21.3%)	339 (25.2%)
≥30 (obesity)	3729 (8.4%)	259 (19.3%)
Smoking during pregnancy		
Missing	3137 (7.1%)	122 (9.1%)
Non-smoker	36,962 (83.4%)	1140 (84.8%)
Smoker	4199 (9.5%)	83 (6.2%)
Household disposable income		
Not registered or <80,000 SEK/year	1811 (4.1%)	63 (4.7%)
Lowest quintile	7955 (18.0%)	232 (17.2%)
Second quintile	8282 (18.7%)	280 (20.8%)
Third quintile	8653 (19.5%)	284 (21.1%)
Fourth quintile	8769 (19.8%)	266 (19.8%)
Highest quintile	8828 (19.9%)	220 (16.4%)
Maternal educational level		
Primary (9 yrs)	5910 (13.3%)	149 (11.1%)
Secondary (12 yrs)	19,097 (43.1%)	647 (48.1%)
Post-secondary (>12 yrs)	19,291 (43.5%)	549 (40.8%)
Maternal region of birth		
Sweden	31,754 (71.7%)	1037 (77.1%)
Nordic countries	928 (2.1%)	19 (1.4%)
EU-27	1858 (4.2%)	55 (4.1%)
Rest of Europe incl Russia	2850 (6.4%)	77 (5.7%)
The Americas	678 (1.5%)	24 (1.8%)
Asia and Oceania	5302 (12.0%)	101 (7.5%)
Africa	928 (2.1%)	32 (2.4%)

**Table 2:** Risk ratios (RR) and 95% confidence intervals (CI) for gestational diabetes and preeclampsia by maternal education and income level.

Preeclampsia (*n* = 1345)					
Maternal Education	No Preeclampsia	Preeclampsia	Model 1 ^a^	Model 2 ^b^	Model 3 ^c^
Primary (9 yrs)	6098	149	0.87 (0.72, 1.06)	1.09 (0.85, 1.39)	1.16 (0.89, 1.50)
Secondary (12 yrs)	19,522	647	1.19 (1.05, 1.34)	1.24 (1.09, 1.41)	1.23 (1.08, 1.41)
Post-secondary (>12 yrs)	19,653	549	1.00 (Reference)	1.00 (Reference)	1.00 (Reference)
Maternal income quintile					
Not registered or <80,000 SEK/year	1846	63	1.39 (1.05, 1.85)	1.17 (0.83, 1.65)	1.31 (0.91, 1.87)
Lowest	8178	232	1.16 (0.96, 1.40)	1.07 (0.86, 1.34)	1.25 (0.99, 1.59)
Second	8496	280	1.35 (1.13, 1.61)	1.32 (1.07, 1.62)	1.36 (1.10, 1.68)
Third	8822	284	1.32 (1.10, 1.57)	1.19 (0.99, 1.45)	1.22 (1.00, 1.48)
Fourth	8935	266	1.22 (1.02, 1.46)	1.13 (0.89, 1.33)	1.14 (0.90, 1.33)
Highest	8996	220	1.00 (Reference)	1.00 (Reference)	1.00 (Reference)

^a^ Model 1. Unadjusted model. ^b^ Model 2. Adjusted for maternal age at childbirth, BMI, parity, and smoking during pregnancy. ^c^ Model 3. Model 2 additionally adjusted for region of birth.

**Table 3:** Risk ratios (RR) and 95% confidence intervals (CI) for gestational diabetes and preeclampsia by maternal region of origin.

Women Total, *n* = 46,618	Reference Women	Preeclampsia	Model 1 ^a^	Model 2 ^b^	Model 3 ^c^
Sweden	32,252	1037	1.00 (Reference)	1.00 (Reference)	1.00 (Reference)
Nordic countries	944	19	0.63 (0.40, 0.99)	0.61 (0.39, 0.96)	0.68 (0.43, 1.06)
EU-27	1897	55	0.90 (0.68, 1.20)	0.88 (0.66, 1.17)	0.97 (0.73, 1.29)
Rest of Europe incl Russia	2919	77	0.82 (0.65, 1.04)	0.76 (0.59, 0.97)	0.89 (0.69, 1.14)
The Americas	696	24	1.07 (0.70, 1.65)	1.01 (0.66, 1.55)	1.00 (0.65, 1.53)
Asia and Oceania	5580	101	0.56 (0.45, 0.70)	0.54 (0.43, 0.67)	0.60 (0.47, 0.75)
Africa	985	32	1.01 (0.69, 1.49)	0.96 (0.65, 1.43)	1.05 (0.69, 1.58)

^a^ Unadjusted model. ^b^ Adjusted for socio-economic factors: maternal education and household disposable income. ^c^ Adjusted additionally for maternal age at childbirth, BMI, parity, and smoking during pregnancy.

## Data Availability

The datasets generated or analyzed during the current study are not available publicly because they are subject to national data protection laws and restrictions imposed by the ethics committee to ensure privacy of study participants.

## Supplementary Materials

The following supporting information can be downloaded at: https://www.mdpi.com/article/10.3390/ijerph19074080/s1, Table S1: Characteristics of women who were included and excluded, respectively, due to missing data on maternal education, numbers in n and (%).
