# Supplementary material for: Influence of Socio-Economic Factors and Region of Birth on the Risk of Preeclampsia in Sweden
Source: Int J Environ Res Public Health. 2022 Mar 30;19(7):4080. doi: 10.3390/ijerph19074080 (PMC8998104; doi:10.3390/ijerph19074080)
Supplement: Supplementary file 1 [file ijerph-19-04080-s001.zip › ijerph-1612684-supplementary.pdf]

Supplementary Table S1. Characteristics of women who were included and excluded, respectively, due to missing data on maternal education, numbers in n and (%).

	Included women	Excluded women
Women total = 48,681	n = 46618	n = 2063
Maternal age (yrs)		
<25	6537 (14.0%)	950 (46.0%)
25-29	14521 (31.1%)	541 (26.2%)
30-34	16536 (35.5%)	363 (17.6%)
35-39	7611 (16.3%)	163 (7.9%)
≥40	1413 (3.0%)	46 (2.2%)
Pre-pregnancy BMI (kg/m^2)		
Missing	5645 (12.1%)	278 (13.5%)
<18.5 (underweight)	1087 (2.3%)	59 (2.9%)
18.5 - <25 (normal weight)	25664 (55.1%)	1039 (50.4%)
25- < 30 (overweight)	10021 (21.5%)	437 (21.2%)
≥30 (obesity)	4201 (9.0%)	250 (12.1%)
Parity		
1	21851 (46.9%)	844 (40.9%)
2	16034 (34.4%)	550 (26.7%)
≥3	8733 (18.7%)	669 (32.4%)
Maternal region of birth		
Missing		8 (0.1%)
Sweden	33289 (71.4%)	121 (5.9%)
Nordic countries	963 (2.1%)	153 (7.4%)
EU-27	1952 (4.2%)	196 (9.5%)
Rest of Europe incl Russia	2996 (6.4%)	377 (18.3%)
The Americas	720 (1.5%)	31 (1.5%)
Asia and Oceania	5681 (12.2%)	973 (47.2%)
Africa	1017 (2.2%)	204 (9.9%)
Smoking during pregnancy		
Missing	3327 (7.1%)	135 (6.5%)
Non-smoker	38916 (83.5%)	1701 (82.5%)
Smoker	4375 (9.4%)	227 (11.0%)
Household disposable income[c]		
Not registered or <80,000 SEK/year	1909 (4.1%)	492 (23.8%)
Lowest quintile	8410 (18.0%)	846 (41.0%)
2nd quintile	8776 (18.8%)	479 (23.2%)
3rd quintile	9106 (19.5%)	151 (7.3%)
4th quintile	9201 (19.7%)	55 (2.7%)
Highest quintile	9216 (19.8%)	40 (1.9%)
Maternal education[d]		
Missing		2058 (99.8%)
Primary (9 yrs)	6247 (13.4%)	4 (0.2%)
Secondary (12 years)	20169 (43.3%)	1 (0.0%)
Post-secondary (>12 yrs)	20202 (43.3%)	
Gestational diabetes	1027 (2.2%)	68 (3.3%)
Preeclampsia	1345 (2.9%)	38 (1.8%)
